# Correspondence

**Published:** 1914-03

**Authors:** S. I. Gubb

**Affiliations:** Late Editor of the Medical Press and Circular, London


Dear Mr. Editor: 31 January, 1914.

I note that the January number of your journal contains an abstract of an article (page 399) on "The Treatment of the Præ-Tuberculous Stage of Consumption," to which my name is appended as author. I beg to inform you that my name has been used without my authority and I distinctly repudiate any responsibility therefor. The opinions embodied in that article are not my opinions, and I have had no experience of the remedy referred to. I trust you will make this clear to your readers.

Yours faithfully,

S. I. GUBB, M. D.,

Late Editor of the Medical Press and Circular, London.

